# Microencapsulated fluorescent pH probe as implantable sensor for monitoring the physiological state of fish embryos

**DOI:** 10.1371/journal.pone.0186548

**Published:** 2017-10-18

**Authors:** Anton Gurkov, Anton Sadovoy, Ekaterina Shchapova, Cathleen Teh, Igor Meglinski, Maxim Timofeyev

**Affiliations:** 1 Institute of Biology, Irkutsk State University, Irkutsk, Russia; 2 Baikal Research Centre, Irkutsk, Russia; 3 Institute of Materials Research and Engineering, Agency for Science, Technology and Research, Singapore; 4 Institute of Molecular and Cell Biology, Agency for Science, Technology and Research, Singapore; 5 Optoelectronics and Measurement Techniques Laboratory, University of Oulu, Oulu, Finland; Washington State University, UNITED STATES

## Abstract

*In vivo* physiological measurement is a major challenge in modern science and technology, as is environment conservation at the global scale. Proper toxicological testing of widely produced mixtures of chemicals is a necessary step in the development of new products, allowing us to minimize the human impact on aquatic ecosystems. However, currently available bioassay-based techniques utilizing small aquatic organisms such as fish embryos for toxicity testing do not allow assessing in time the changes in physiological parameters in the same individual. In this study, we introduce microencapsulated fluorescent probes as a promising tool for *in vivo* monitoring of internal pH variation in zebrafish embryos. The pH alteration identified under stress conditions demonstrates the applicability of the microencapsulated fluorescent probes for the repeated analysis of the embryo’s physiological state. The proposed approach has strong potential to simultaneously measure a range of physiological characteristics using a set of specific fluorescent probes and to finally bring toxicological bioassays and related research fields to a new level of effectiveness and sensitivity.

## Introduction

Industrially produced chemicals have become widespread in the biosphere due to human activity and can already be found even in deep-water animals [1–2], including invertebrates from the deepest point on the planet—the Mariana Trench [3]. There are over 100 million substances registered in the Chemical Abstract Service, a significant portion of which are widely used in agriculture, industry and by individuals [4], and these substances are further concentrated in rivers and lakes. Due to possible toxicity to different inhabitants of water reservoirs, these chemicals should generally be tested for their biological effects at the cellular to ecosystem levels [5] to prevent or minimize negative impacts on the environment [6]. A number of environmental disasters, such as massive fish deaths in Louisiana in 1950s caused by uncontrolled use of the insecticide endrin in agriculture, exemplify the importance of preliminary ecotoxicological testing [7]. It is worth mentioning that in nature, aquatic organisms are mostly affected by complex mixtures of chemicals, not individual agents [8], which makes the required amount of experimental analyses for realistic toxicity testing enormous.

To accelerate these analyses, bioassays generally applied for environmental risk assessment are mostly based on small organisms such as fish embryos, small crustaceans or algae [9–10] due to their convenient laboratory handling and high reproduction rate. A widely used method of assessing toxicity is to search for morphological abnormalities during embryonic development [11], but this method does not provide the high sensitivity to pathological processes exhibited by screening of internal physiological parameters [12]. However, in the identification of physiological markers, the small size of the used test objects has a significant disadvantage: usually such organisms are equal or smaller than the tissue sample required by the existing methods for measurement of the physiological parameters of interest [13–15]. The situation can be roughly described by the formula “1 organism ≤ 1 sample”. Due to this limitation, researchers must use different individuals at each exposure point, which can increase measurement inaccuracy because of interindividual variability (if 1 organism = 1 sample) or can oversmooth individual-specific reactions (if 1 organism < 1 sample). Moreover, it also increases the number of organisms used for the experimental procedure, which increases the time and cost of the analysis. For these reasons, modern environmental toxicology demands new techniques that can continuously monitor the physiological parameters of a single small organism *in vivo*.

A promising way to overcome this limitation is to apply microencapsulated fluorescent probes for repeated *in vivo* measurements. Encapsulation of fluorescent dyes for *in vivo* applications has two fundamental benefits: it enables ruling out any possible toxicity of the dye itself, and concentration of the probe to a single point to give a strong and easily detectable fluorescent signal. The microcapsules containing the fluorescent probe can be implanted into the organism and serve as artificial biomarkers of physiological parameters; thus, we call them microencapsulated biomarkers (MBMs).

There is a broad variety of commercial fluorescent molecular probes sensitive to various ions and metabolites [16], and a pH-sensitive dye was chosen for this study as a “first pass” of the proposed technique, due to the high importance of this parameter. As a factor that is especially valuable for toxicity assessment, the pH of internal fluids depends on proper functioning of organs such as the liver and kidney—the main targets of the majority of toxicants [17–19]; changes in pH can be used as markers of malfunction in these and other internal organs under intoxication [20–21]. In the current study, the embryo of *Danio rerio*, known as zebrafish, was chosen due to its importance as a “gold-standard” laboratory animal not only for toxicology and environmental sciences [22–23] but also for a broader range of research fields such as developmental biology [24] and neurophysiology [25–26].

The proof-of-concept using encapsulated pH-sensitive fluorescent dyes as intracellular sensors was demonstrated in mammalian cells *ex vivo* [27]. Low cytotoxicity of the microcapsules also was shown [28], and polyethylene glycol-grafted poly-L-lysine (PLL-g-PEG) was suggested as coverage to significantly reduce phagocytosis of the microcapsules by immune cells [29]. Studies of MBM biocompatibility after injection into the pericardium were performed utilizing zebrafish embryos and showed no effect of MBMs on embryo development [30]. More recently, it was shown that MBMs can be successfully used to identify physiological changes *in vivo* in adult crustaceans and fishes under stress conditions [31–32]. However, similar measurements with smaller organisms such as fish embryos, require more advanced and sophisticated procedures. Therefore, in the framework of further development of the MBM approach, we report the application of microencapsulated fluorescent probes for measurement of pH variation in zebrafish embryos *in vivo* to monitor the physiological state of the embryo under stressful conditions.

## Materials and methods

### Preparation of pH-sensitive MBMs

**Fig 1:**
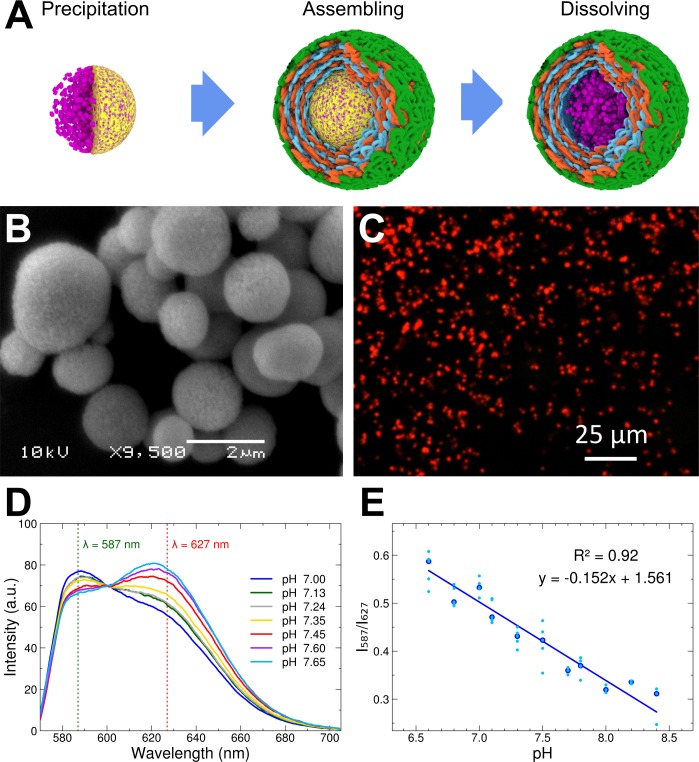
Description of pH-sensitive MBMs used. (a) General scheme of preparation of MBMs using the LbL method: co-precipitation of SNARF-1-D (purple) into porous CaCO_3_ cores (yellow); LbL assembly of microcapsule shell around the cores (only three layers of negatively charged polymer, three layers of positively charged polymer and final biocompatible layer are depicted); and dissolution of cores. (b) SEM image of porous CaCO_3_ cores with incorporated SNARF-1-D. (c) Prepared MBMs under confocal laser scanning microscope LSM 700. (d) Examples of fluorescence spectra of microencapsulated SNARF-1-D with varying pH. (e) Calibration curve of MBMs containing SNARF-1-D with varying pH was built based on median values (emphasized by larger dark blue points); original values are also depicted (smaller light blue points).

The developed MBMs consist of a semipermeable multilayer shell prepared by layer-by-layer (LbL) assembly of oppositely charged polyelectrolytes (Fig 1A). SNARF-1 conjugated with dextran of molecular weight 70,000 Da (SNARF-1-D; Invitrogen, D-3304) was chosen as the fluorescent probe to measure pH due to its good sensitivity at pH 6–9. Conjugation of the probe to dextran is required to trap the sensor inside the semipermeable shell.

SNARF-1-D was encapsulated according to previous recommendations [27, 29–30] with modifications (Fig 1A). First, SNARF-1-D was co-precipitated into porous cores of CaCO_3_ by mixing 2 ml of 0.25 mg/ml SNARF-1-D and a 4 mg/ml dextran sulfate solution with 0.615 ml of a 1 M Na_2_CO_3_ solution and 0.615 ml of a 1 M CaCl_2_ solution under strong stirring (Fig 1B). Dextran sulfate was added to give CaCO_3_ cores a more regular form. After agitating for 15 s at room temperature, the mixture was quickly washed in deionized water three times to stop precipitation and treated with ultrasound to destroy aggregates. The CaCO_3_ cores were visualized under a JSM 6360LA scanning electron microscope (JEOL, USA).

The CaCO_3_ cores were then covered with 10 layers of oppositely charged polymers: positive poly(allylamine hydrochloride) (PAH; Aldrich, 2832315) and negative poly(sodium 4-styrenesulfonate) (PSS; Aldrich, 243051). After each layer, the cores were washed several times in deionized water and treated with ultrasound every two layers. To enhance the biocompatibility of the MBMs, they were covered with polyethylene glycol-grafted poly-L-lysine (PLL-g-PEG; *g* = 3.5) as the outmost layer [30]. Finally, the CaCO_3_ templates were dissolved in EDTA solution (pH 7.0) to obtain a semipermeable polyelectrolyte shell with the structure (PSS/PAH)_5_-PLL-g-PEG trapping SNARF-1-D inside (Fig 1C). The prepared MBMs had a median size of approximately 1.3 μm.

Example spectra of the microencapsulated SNARF-1-D at different pHs (Fig 1D) were obtained with an Eclipse Ti-U fluorescent microscope (Nikon, Japan) coupled with the Sherlock 300 spectrometer (Andor, USA). Fluorescence was excited by light of approximately 563 nm.

### Calibration of MBMs to various pHs

The prepared MBMs must be calibrated to pH before use. For this purpose MBMs were dispersed in a series of buffer solutions. Calibration is best performed using the same microscope that will be later used for *in vivo* pH assessment; we used an inverted LSM 700 (Carl Zeiss, Germany) confocal microscope (S1 Fig). Laser light at 555 nm was used to excite encapsulated SNARF-1-D with sequential emission signal acquisition in the green channel (587 nm) and the red channel (627 nm) for further ratiometric pH measurements. Images of MBMs in buffers and inside animals consist of three channels: a 587-nm channel, a 627-nm channel and a white light channel.

The obtained images were analyzed using the Fiji image processing software package (www.fiji.sc). The brightest image (green or red channel) was chosen to make a mask by thresholding the image. The mask was applied to the images in the green and red channels to extract a fluorescent signal from the MBMs. Then, total pixel intensity was measured for every MBM area in both the green and red channels, and the ratio I_587_/I_627_ between the channels was calculated. The relationship between median I_587_/I_627_ and the pH of the buffered media was well approximated using the following linear function: pH = –6.11 * I_587_/I_627_ + 10.07. The obtained calibration curve is displayed in Fig 1E.

### Injections of MBMs into zebrafish embryos

Wild-type zebrafish (strain AB) were used as the vertebrate model to assess physiological pH *in vivo*. Zebrafish was maintained in the Zebrafish Facility at the Institute of Molecular and Cell Biology, A*STAR, Singapore. Fish-based experiments were performed in accordance with a protocol approved by the Institutional Animal Care and Use Committee (IACUC) in Biological Resource Center in Biopolis, Singapore (IACUC #120787). All embryos were grown and maintained in egg water (0.6 g/l of aquarium salt in water purified with a reverse osmosis system) at 28.5°C.

Delivery of the MBMs to the intestine of 72 hpf zebrafish embryos (10 animals) via microgavage was performed as stated [33]. Injection of the MBMs to the brain ventricle was performed in 24 hpf zebrafish embryos. To ensure the optical translucence of zebrafish embryos for confocal imaging, egg water with 1-phenyl-2-thiourea was added to 22 hpf embryos. Zebrafish embryos were removed from their chorions before the microinjection experiments. The positions of “to be” injected zebrafish embryos were adjusted in molten 1% low melting agarose (at approximately 30°C) with the brain ventricle facing upward. For the injection, we used a glass pipet (tip diameter adjusted to 0.1 mm) pulled from a thin-walled glass microcapillary tube (outer diameter of 1 mm and inner diameter of 0.75 mm; Sutter Instruments). Pressured injection of approximately 5 nl of a suspension of MBMs in normal saline solution (approximately 10 microcapsules per 1 nl) was achieved using a pico-injector (PLI-100, Harvard Apparatus). A previous study demonstrated no effect of microcapsules injected into the circulatory system of zebrafish embryos on their survival or development and no blood flow disruptions [30].

### pH measurements *in vivo* with MBMs

Images of MBMs inside zebrafish embryos were acquired using an inverted LSM 700 confocal microscope (S1 Fig). The pH measurements were performed *in vivo* with MBMs injected into the forebrain ventricle of zebrafish embryos. Ratiometric analysis of the MBM fluorescence intensity in the 587 nm and 627 nm channels was performed as described for the calibration of MBMs to various pHs. The pH in the cerebrospinal fluid was derived after fitting the I_587_/I_627_ intensity ratio to the obtained linear calibration curve (Fig 1B).

The cerebrospinal fluid pH was measured under the control conditions and right after acute heat shock at 50°C for five minutes. The pH before and after the temperature treatment was evaluated in the same clusters of MBMs in the forebrain ventricle to ensure the highest accuracy measurements. The statistical significance of differences between the experimental groups was tested with a two-sided Mann-Whitney U test in the statistical software R (www.r-project.org) with the additional package *coin* [34].

## Results and discussion

The applied MBMs (Fig 1A–1C) are based on the pH-sensitive fluorescent dye SNARF-1, which was encapsulated in a semipermeable polyelectrolyte shell using the LbL technique [27, 30]. To keep SNARF-1 inside the shell, the dye was encapsulated in the form of a conjugate with dextran (SNARF-1-D). The shell is only permeable to low-weight molecules, allowing the fluorescent probe to remain sensitive to the environment; however, it traps the dye conjugated with the polymer inside the microcapsule. MBMs were made biocompatible by covering them with a polymer containing polyethylene glycol, which has high resistance to protein adsorption and significantly reduces friction [35–37]. Due to this coating, MBMs should not be recognized by the immune system and should easily move in the internal media of an animal.

SNARF-1 was excited by approximately 560 nm light to yield a fluorescence spectrum with two peaks at approximately 587 and 627 nm. The ratio between the peaks is sensitive to pH (Fig 1D), and thus, the dye may serve as a self-sufficient selective sensor to pH with the best sensitivity at pH 6–9 [16], which perfectly fits measurements in the physiological pH range. The ratio between the spectral peaks I_587_/I_627_ of SNARF-1 inside MBMs has an apparent linear response to the pH of the media and was calibrated in a series of buffers from pH 6.6–8.4 (Fig 1E) before further *in vivo* measurements under a confocal microscope (S1 Fig). The response of microencapsulated SNARF-1 to pH is known to be highly reversible [38], which allow using this probe for prolonged pH monitoring.

**Fig 2:**
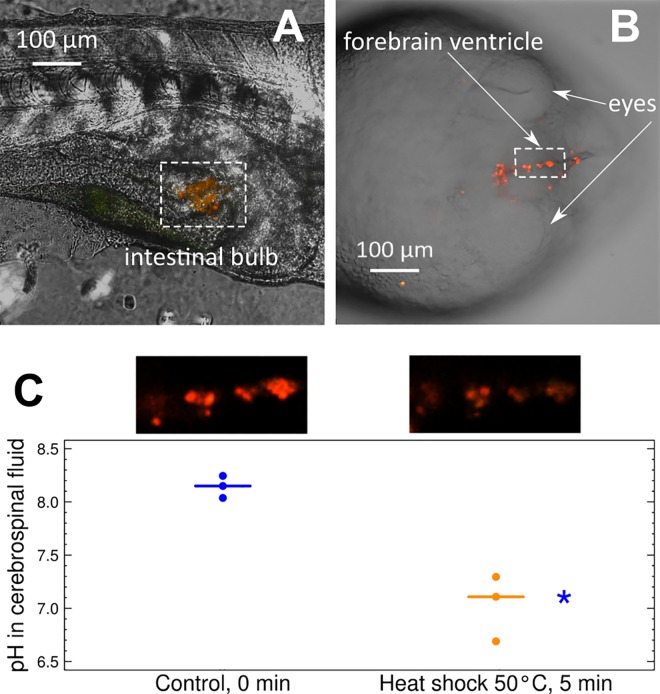
Visualization of MBMs and pH measurements in zebrafish embryos. (a) Images of MBMs in the intestine of a zebrafish embryo, combined green (587 nm) and red (627 nm) channels. (b) Images of MBMs in brain ventricle of a zebrafish embryo, combined green (587 nm) and red (627 nm) channels. (c) pH in cerebrospinal fluid monitored by MBMs under control and heat shock conditions with respective original images of MBMs in the brain ventricle of the same individual. Blue indicates control conditions; orange indicates heat shock exposure. * indicates a statistically significant difference from the control with *p*-value < 0.05.

To test the possibility of acquiring the fluorescent signal from MBMs inside zebrafish embryos, a series of embryos were microgavaged with many MBMs. The fluorescence of MBMs was easily identifiable in the digestive system of all the microgavaged embryos (Fig 2A). MBMs spread and moved freely along the intestine of the embryos over three hours of observation.

In the next step, we tested the possibility of acquiring signal from a small amount of MBMs injected into the internal fluids of the organism, particularly inside the brain ventricle of a zebrafish embryo (Fig 2B). The brain ventricle of fish embryos is a unique site for simple introduction of optical sensors into an organism without any effects of the injection on survival, development or behavior of the embryos [39]. The fluorescent signal was recognizable even from clusters of just a few MBMs or individual MBMs inside the forebrain ventricle of the embryos, and the intensity ratio I_587_/I_627_ corresponded to a pH range of 8.04–8.25, according to the obtained calibration curve. The measured pH of the cerebrospinal fluid was in good concordance with previous data. Generally, the pH of the cerebrospinal fluid should be similar to blood pH [40], which lies in range of 7.7 to 8.0 for the adults of most fish species [41]. The pH values measured by MBMs may be slightly more alkaline due to small inaccuracies in the measurements. Additionally, in the case of zebrafish embryos during early stages of development, interstitial pH is approximately 7.8–8.3 [42]. The embryos currently used for injections are at the late stages of embryonic development, but may still have more alkaline blood and cerebrospinal fluid than adults.

To test the sensitivity of the MBMs to physiological changes in pH, the embryos were subjected to acute heat shock at 50°C for five min (Fig 2C). Such exposure should lead to severe damage or death of the animal, and it was applied to generate the highest possible change in physiological pH. After the temperature treatment, the readout of the MBMs indicated a statistically significant decrease (*p*-value = 0.049) in pH to approximately 6.7–7.3. This shift is likely related to a switch to anaerobic metabolism under the strong heat shock and to accumulation of lactic acid in tissues and blood of the organism. A variety of stressful conditions (and heat shock) are known to cause a decline in oxygen-related energy production in mitochondria, while anaerobic energy production (with lactate as the final metabolite) becomes intense to compensate the energy demand [43–44]. Accumulation of lactate, indicated by the observed acidification of the internal fluids of the embryos, reveals significant physiological disturbance under the applied heat shock.

The obtained results demonstrate the possibility of monitoring physiological parameters of the same fish embryo over time with a common confocal microscope (or even a simple fluorescent microscope), which could significantly improve the current bioassays used for toxicity testing. Moreover, the technique suggested in the current work has methodological advantages over the widely applied procedures for identification of physiological parameters. Methods such as real-time PCR, gel electrophoresis of proteins, biochemical enzymatic techniques and others require long sample preparation procedures, while the injection of MBMs takes much less time, after which *in vivo* pH monitoring is readily possible.

Importantly, the proposed technology of MBMs has a number of advantages for physiological measurements over other existing techniques for immobilization of fluorescent probes into polymeric nano- and microbeads (S1 Table) [45–62]. Unlike nanosensors, MBMs are large enough to observe and obtain a signal from even a single microcapsule with an optical microscope, and, unlike hydrogel microbeads, MBMs can roll up (similar to red blood cells) to move along the smallest capillaries without disruption of the blood flow [30] when injected into the circulatory system. Furthermore, MBMs are the only type of immobilized sensors that can be biodegradable and can incorporate different enzymes into the sensor, which significantly increases the range of measurable parameters.

Finally, MBMs allow the use of combinations of probes for different physiological parameters. The microcapsules trapping different fluorescent dyes can “mask” their possible toxicity to the analyzed organism, which removes concerns about the toxicity of many different dye mixtures and may hasten the development of new bioassays.

## Conclusions

In the current study, we introduced a microscopy-based approach that offers significant advances for monitoring physiological characteristics in fish embryos *in vivo*. The brain ventricle was proposed as a unique site for simple and harmless implantation of MBMs into fish embryos, and the implanted MBMs showed sensitivity to pH variation in zebrafish embryo under heat shock stress conditions. In contrast to currently available biochemical and molecular techniques, the use of MBMs allowed repeated measurements in the same individual and monitoring of the physiological state of the organism. In the current study, we focused on pH-sensitive MBMs, but with further development, we will increase the range of MBMs sensitive to various physiological parameters. This feature allows more ambitious and comprehensive *in vivo* monitoring of the states of fish embryos, with a final aim to bring toxicological bioassays, and ecotoxicology- and ecophysiology-related research to a new level of effectiveness and sensitivity.

## Supporting information

S1 Fig General scheme of optical system in confocal laser scanning microscope LSM 700 used for *in vivo* pH measurements with implanted MBMs. (TIF)

S1 Table Comparison of different techniques for the immobilization of fluorescent probes in polymeric nano/microcapsules and nano/microbeads/fibers for *in vivo* applications [45–62]. (PDF)
