# Supplementary figures and images for: Microencapsulated fluorescent pH probe as implantable sensor for monitoring the physiological state of fish embryos
Source: PLoS One. 2017 Oct 18;12(10):e0186548. doi: 10.1371/journal.pone.0186548 (PMC5646854; doi:10.1371/journal.pone.0186548)

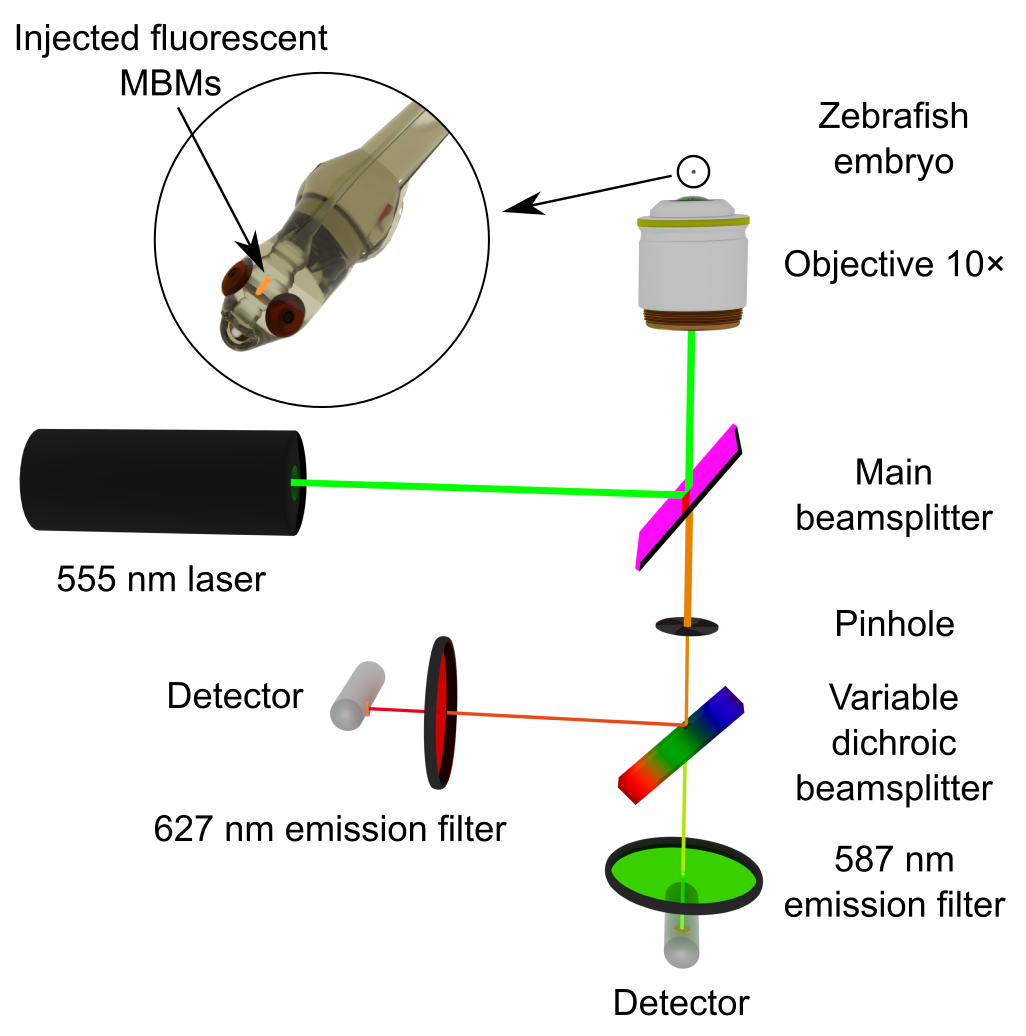

Supplement: S1 Fig — (TIF) [file pone.0186548.s001.tif]
