# Supplementary material for: Microencapsulated fluorescent pH probe as implantable sensor for monitoring the physiological state of fish embryos
Source: PLoS One. 2017 Oct 18;12(10):e0186548. doi: 10.1371/journal.pone.0186548 (PMC5646854; doi:10.1371/journal.pone.0186548)
Supplement: S1 Table — (PDF) [file pone.0186548.s002.pdf]

**Features**	**Immobilization techniques as named by the authors**		
	**Microencapsulated biomarkers (MBMs)**	**Nanosensors / nanooptodes** [52-57]	**Hydrogel microbeads / fibers** [59-60]
Type of immobilization	LbL-assembled semipermeable polyelectrolyte microcapsule	Particles of poly(vinyl) chloride plasticized by tetrahydrofuran	Polyacrylamide gel
Co-localization of different fluorescent probes	Allows co-encapsulation of several probes [45]	Combination of different probes in one sensor is possible but limited, as some sensors are based on the same fluorescent probe in combination with different selective ionophores	Only sensors to glucose are developed, but potentially other probes may be added
Form	Hollow sphere	Ball or fiber	Ball or fiber
Diameter	~ 0.5-30 µm, adjustable [46-47]	Nanosensors: ~ 0.02-0.40 µm, adjustable;<br>Fibers: ~ 0.2-0.5 µm	Microbeads: 100-150 µm;<br>Fibers: 500-1000 µm
Hydrophilic/hydrophobic shell	Hydrophilic	Hydrophobic	Hydrophilic
Biocompatible coverage	Yes	Yes	Yes
Possibility to make the shell biodegradable	Yes [48]	No	Polyacrylamide is hardly biodegradable [61], but other hydrogels may allow biodegradation
Toxicity of shell components	No significant toxicity identified [49-50]	Tetrahydrofuran has low to moderate toxicity [58]. Moreover, functional molecules (ionophores, dyes, *etc.*) with potential toxicity are not anchored in the hydrophobic core and may transfer to lipid fractions inside organism	Acrylamide monomer has neurotoxicity [62], but it is declared to be washed from the gel
Possibility to incorporate enzymes and fluorescent proteins in native state	Yes [51]	Only on surface of sensor	Likely yes, but not shown
